# Everolimus pharmacokinetics and exposure-response relationship in Japanese patients with advanced breast cancer

**DOI:** 10.3389/fphar.2022.984002

**Published:** 2022-09-15

**Authors:** Masaki Hirabatake, Tomoyuki Mizuno, Hironori Kato, Tohru Hashida

**Affiliations:** ^1^ Department of Pharmacy, Kobe City Medical Center General Hospital, Kobe, Japan; ^2^ Division of Clinical Pharmacology, Cincinnati Children’s Hospital Medical Center, Cincinnati, OH, United States; ^3^ Department of Pediatrics, University of Cincinnati College of Medicine, Cincinnati, OH, United States; ^4^ Department of Breast Surgery, Kobe City Medical Center General Hospital, Kobe, Japan

**Keywords:** everolimus, advanced breast cancer, pharmacokinetics, blood concentration, efficacy, adverse events

## Abstract

**Background:** Everolimus is one of the key drugs for the treatment of advanced breast cancer. The optimal target concentration range for everolimus therapy in patients with breast cancer has not yet been established. This study aimed to characterize everolimus pharmacokinetics (PK) and determine the relationship between blood concentration and efficacy as well as adverse events in patients with breast cancer.

**Methods:** This was a prospective, observational PK study. Patients receiving everolimus between November 2015 and November 2018 at our hospital were enrolled in this study. The whole blood samples for the everolimus assay were collected at least two weeks after initiation of treatment or the last everolimus dose change. PK parameters were estimated using Bayesian analysis. Statistical differences in everolimus trough concentrations between patient cohorts were assessed using the Mann–Whitney test. Progression-free survival was assessed using the Kaplan-Meier method and the log-rank test.

**Results:** Eighteen patients were enrolled in the study. The median follow-up period was 35 months. The most frequently observed adverse event was stomatitis (all grade 94%). There was high inter-individual variation in PK parameters such as clearance [range: 5.1–21.3 L/h/70 kg and co-efficient of variation (CV): 38.5%] and volume of distribution of the central compartment (range: 9.9–103.6 L/70 kg and CV: 57.8%). The trough concentrations at dose-limiting toxicities were significantly higher than trough concentrations in the absence of these toxicities (*p* = 0.0058). Progression-free survival was significantly longer in the 10–20 ng/ml group than in the other groups (*p* = 0.0078).

**Conclusion:** This study characterized the everolimus PK parameters in Japanese patients with breast cancer. High everolimus exposure was found to be associated with poor tolerability. Based on our data, trough concentrations in the range of 10–20 ng/ml may be associated with prolonged progression-free survival. Thus, determining the blood concentration of everolimus and subsequent dose adjustments will potentially reduce side effects and enhance the therapeutic effect in Japanese patients with advanced breast cancer.

## Introduction

Endocrine therapy is used as a first-line treatment for advanced or metastatic breast cancer in hormonally sensitive patients, considering there is no imminent life-threatening risk (Hortobagyi, 1998; Gradishar et al., 2017). When endocrine therapy is unsuccessful, the treatment regimen is changed to chemotherapy, involving anthracyclines or taxanes. Everolimus and exemestane combination therapy is one of the key treatment strategies used before transitioning to chemotherapy (Yardley et al., 2013; Jerusalem et al., 2016; Im et al., 2021). Everolimus, an inhibitor of the mammalian target of rapamycin (mTOR) (Schuler et al., 1997; Dennis et al., 1999; Nashan, 2002), is used not only for treating inoperable or recurrent breast cancer but also for suppressing rejection in organ transplantation and for the treatment of tuberous sclerosis complex (Eisen et al., 2003; Tedesco Silva et al., 2010; French et al., 2016; Jeng et al., 2018). However, the adverse effects caused by treatment with everolimus are more frequent in breast cancer patients than in organ transplantation patients (Mjörnstedt et al., 2012; Pritchard et al., 2013) and are particularly prevalent among Japanese patients (Hattori et al., 2014; Ito et al., 2015). For example, the incidence of stomatitis, the most characteristic adverse event of everolimus, is approximately 10% in renal transplant or tuberous sclerosis complex, but 20%–50% when used in patients with breast cancer (Alexopoulos et al., 2022; Ruíz-Falcó Rojas et al., 2022; Tedesco-Silva et al., 2022). In the BOLERO-2 study, the incidence of stomatitis in Japanese breast cancer patients was 88%. Such frequently caused complications associated with serious adverse events hinder the treatment process, ultimately causing treatment termination.

For organ transplantation, the everolimus dose is individualized based on blood concentration monitoring, to achieve the recommended blood trough concentration (Shipkova et al., 2016). For example, the suggested target trough concentration for everolimus administered to kidney transplant patients is 6–10 ng/ml (calcineurin-inhibitor-free regimen). For tuberous sclerosis, the suggested target trough concentration is 5–15 ng/ml (Combes et al., 2018). While the target concentration range for everolimus therapy has been well characterized in these patient populations, the optimal target concentration in breast cancer treatment has not yet been established owing to a lack of information on the relationship between exposure and response. The present study aimed to characterize everolimus pharmacokinetics (PK) and the relationship between blood concentration and efficacy as well as adverse events in patients with advanced or metastatic breast cancer.

## Materials and methods

### Study design

This was a prospective, observational PK study. Patients with advanced or metastatic breast cancer who received everolimus between November 2015 and November 2018 at Kobe City Medical Center General Hospital were enrolled in this study. The main inclusion criteria included the following: age ≥20 years; Eastern Cooperative Oncology Group (ECOG) performance status (PS): 0 or 1; and adequate bone marrow, liver, and renal function. The main exclusion criteria were interstitial pneumonia or pulmonary fibrosis, HBsAg positive, and psychiatric or psychological symptoms that made it difficult to participate in the study. Every participant provided written informed consent. This study was conducted in accordance with the Declaration of Helsinki and approved by the Institutional Review Board, under Approval Number zn151103.

Based on the physician’s decision, the patients were prescribed a starting dose of either 5 or 10 mg everolimus once daily. The everolimus dose was reduced when a patient experienced adverse events higher than grade 2. The first reduced dose was 5 mg once daily, followed by the second reduced dose of 2.5 mg once daily or 5 mg every alternate day. In addition, everolimus doses could be increased if adverse events improved to grade 1 or less, or deemed inefficacious. The adverse events were evaluated weekly for the first month and then every 1–4 weeks, according to the Common Terminology Criteria for Adverse Events Ver. 4.0.

### Pharmacokinetic sampling and everolimus assay

Whole blood samples for the everolimus assay were collected at least two weeks after initiation of treatment or after the last everolimus dose change. Blood samples were collected at pre-dose, 1, 4, and 8 h after the everolimus dose. Blood everolimus concentrations were measured using a latex agglutination turbidimetric immunoassay (Mori et al., 2014) for the samples collected during November 2015 to September 2016 (*n* = 10) and using electrochemiluminescence immunoassay (Blackburn et al., 1991) for the samples collected during October 2016 to November 2018 (*n* = 8). Since these two techniques are highly correlated, the measured results were considered to be the same (r = 0.972) (Sasano et al., 2016).

### Pharmacokinetic analysis

PK parameters such as clearance (CL) and volume of distribution were estimated using Bayesian analysis in MW/Pharm++ software (Mediware, Prague, Czech Republic) (Fuchs et al., 2013). The two-compartment model parameter estimates reported by de Wit et al. (2016) were used as the Bayesian priors: 20.3 L/h (CV: 38.1%) for clearance (CL), 29.1 (CV: 87.3%) for the volume of distribution of the central compartment (V1), 60 L/h for intercompartmental clearance (Q), 475 L for the volume of distribution of the peripheral compartment (V2), and 0.643 h^−1^ for absorption rate constant (Ka). The same CV% for CL (38.1%) and V1 (87.3%) were applied to Q and V2, respectively as no inter-individual variabilities were reported for these parameters. Ka was fixed for all analyses due to the limited data to characterize the absorption. The PK parameters were allometrically scaled to body weight to account for the effect of body size differences according to the following formula:

$$Pi=Ppop\times {\left(\frac{BWi}{BWstandard}\right)}^{power}$$

where P_i_ was the estimated PK parameter for individual i, P_pop_ was the typical population value of the PK parameters. BW_standard_ was the standard body weight of 70 kg. The power coefficient was 0.75 for CL and intercompartment clearance and 1 for volume of distribution of the central compartment and the peripheral compartment.

Everolimus concentrations at steady-state or at the time of occurrence of dose-limiting toxicity was estimated using the Bayesian-estimated PK parameters of the individual patient.

### Statistical analyses

Statistical differences in everolimus trough concentrations, doses and body weights between patient cohorts were assessed using the Mann–Whitney test. Progression-free survival (PFS) was assessed using the Kaplan-Meier method and the log-rank test. All statistical analyses were performed using GraphPad Prism version 7.03 (San Diego, CA). *p* < 0.05 was considered statistically significant.

## Results

### Patient characteristics and clinical outcomes

Eighteen patients were enrolled in this study. The median follow-up period was 35 months (8–60 months) (cutoff date was 11 August 2019). Table 1 shows the baseline characteristics of the patients. The PS of all patients was 0. The starting dose of everolimus was 10 mg in 12 patients and 5 mg in six patients. Within 33 weeks after the initiation of treatment, eight of the twelve patients who were prescribed 10 mg everolimus required treatment suspension or dose reduction owing to the occurrence of adverse events. Similarly, five of the six patients who were prescribed 5 mg everolimus required treatment suspension or dose reduction within the first 40 weeks of treatment. In addition to exemestane (*n* = 18), gastric mucoprotectants (*n* = 11), vitamins including active vitamin D preparations (*n* = 10), analgesics (*n* = 9), antibacterial agents (*n* = 9), and HMG-CoA reductase inhibitors (*n* = 8) were the primary concomitant drugs. A CYP3A4 inhibitor, clarithromycin 200 mg twice daily for 7 days, was administered to 3 patients during the everolimus treatment period. No patients received CYP3A4 inducers. The most frequently observed adverse events were stomatitis, rash, and hypertriglyceridemia (Table 2).

**TABLE 1:** Baseline patient characteristics.

Age, years	Median (range)	66 (42–85)
Body weight, kg	Median (range)	51.8 (39.0–67.8)
Number of metastatic sites, n (%)	1	8 (44.4)
	2	5 (27.8)
	≧3	5 (27.8)
ECOG performance status, n (%)	0	18 (100)
Number of previous chemotherapy lines in advanced setting, n (%)	1	2 (11.1)
	2	3 (16.7)
	3	6 (33.3)
	≧4	7 (38.9)
Everolimus initial dose, n (%)	10 mg/day	12 (66.7)
	5 mg/day	6 (33.3)
Everolimus dose at PK study, n (%)	10 mg/day	7 (38.9)
	5 mg/day	6 (33.3)
	5 mg/2 days or 2.5 mg/day	5 (27.8)

ECOG, eastern cooperative oncology group.

**TABLE 2:** Most common and grade ≧3 adverse events.

	All patients (*n* = 18)					Initial dose: 10 mg/day (*n* = 12)					Initial dose: 5 mg/day (*n* = 6)				
	Grade					Grade					Grade				
Adverse event, n	All	1	2	3	4	All	1	2	3	4	All	1	2	3	4
Mucositis oral	17	7	10	0	0	11	5	6	0	0	6	2	4	0	0
Rash	14	11	3	0	0	10	8	2	0	0	4	3	1	0	0
Hypertriglyceridemia	12	8	4	0	0	9	6	3	0	0	3	2	1	0	0
High cholesterol	11	9	2	0	0	7	5	2	0	0	4	4	0	0	0
Hypokalemia	9	6	1	1	1	6	4	1	0	1	3	2	0	1	0
Platelet decrease	9	9	0	0	0	7	7	0	0	0	2	2	0	0	0
Malaise	7	6	1	0	0	5	5	0	0	0	2	1	1	0	0
Neutrophil count decreased	6	2	3	1	0	6	2	3	1	0	0	0	0	0	0
Epistaxis	6	6	0	0	0	4	4	0	0	0	2	2	0	0	0
Hyperglycemia	5	4	1	0	0	3	2	1	0	0	2	2	0	0	0
Diarrhea	5	1	4	0	0	4	1	3	0	0	1	0	1	0	0
Dysgeusia	5	4	1	0	0	3	3	0	0	0	2	1	1	0	0
Interstitial lung disease	5	3	2	0	0	4	3	1	0	0	1	0	1	0	0

### PK analysis

The PK data was collected at steady-state at least 2 weeks after the start of everolimus treatment or the last dose change. The doses of everolimus at the time of PK blood sampling were 10, 5, and 2.5 mg/day for seven, six, and two patients, respectively, and 5 mg every alternate day for three patients. The dose-adjusted trough concentrations were within the range of 1.3–6.5 ng/mL/mg/day. Table 3 summarizes the everolimus PK parameter estimates generated using the Bayesian estimation. There was high inter-individual variation in PK parameters, such as CL [range: 5.1–21.3 L/h/70 kg and coefficient of variation (CV): 38.5%] and volume of distribution of the central compartment (range: 9.9–103.6 L/70 kg and CV: 57.8%).

**TABLE 3:** Everolimus pharmacokinetic parameter estimates.

	Mean	SD
CL (L/h/70 kg)	11.7	4.5
V1 (L/70 kg)	54.0	31.2
Q (L/h/70 kg)	42.2	11.4
V2 (L/70 kg)	309.4	144.9

CL, clearance; V1 = central volume of distribution; Q, intercompartmental clearance; V2, peripheral volume of distribution; SD, standard deviation.

Steady-state everolimus trough concentrations at the starting dose, in each patient, were approximated using the Bayesian-estimated PK parameters. The steady-state trough concentrations were between 13.3 and 67.6 ng/ml and 11.3–29.6 ng/ml in the 12 patients on 10 mg starting dose and in the six patients on 5 mg starting dose, respectively. In all patients, everolimus trough concentrations were above the target trough concentration range of 6–10 ng/ml suggested for patients with kidney transplants.

### Association between everolimus exposure and toxicity

Dose-limiting toxicities (DLTs) were observed in 13 patients during the 10 months of the study period. The main DLTs were stomatitis (Grade 2; *n* = 5), interstitial pneumonia (Grade 2; *n* = 2), and hypokalemia (Grade 4; *n* = 1 and Grade 2; *n* = 1). The median estimated trough concentration in the 13 patients at the time of the DLTs was 19.0 ng/ml (range: 11.3–64.6 ng/ml). In these patients, the median estimated steady-state trough concentration was 8.3 ng/ml (range: 5.7–24.2 ng/ml) when the dose was reduced and maintained without DLT. The trough concentration (median and range) in the five patients who did not experience DLTs was 19.0 ng/ml (12.8–33.2 ng/ml).

**FIGURE 1:**
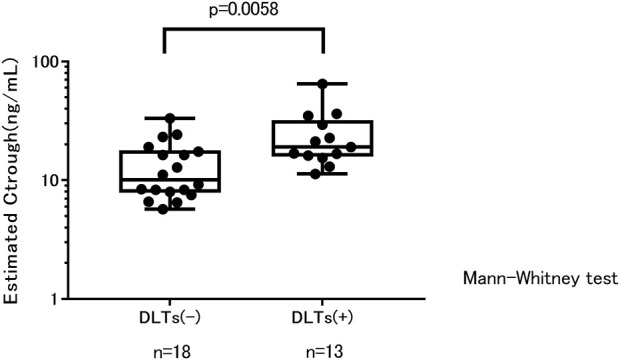
Trough concentration estimates and treatment tolerability. C_trough_, trough concentration; DLTs, dose-limiting toxicities.

We defined the trough concentration in the DLT-group as the trough concentration at which time patients did not show DLT. This includes patients who did not have any DLT (*n* = 5) and patients who had DLT (*n* = 13) at the initial dose but reduced the dose and continued treatment without grade ≥2 adverse events. Trough concentrations were significantly higher when patients showed DLT (*n* = 13) than when patients did not show DLT (*n* = 18) (Figure 1).

The DLT + group received a significantly higher dose than the DLT-group (mean ± SD: 8.1 ± 2.4 mg vs. 5.4 ± 2.8 mg, *p* = 0.0099). Body weight was not significantly different between the DLT+ and DLT-groups (mean ± SD: 51.4 ± 6.0 kg vs. 52.2 ± 7.0 kg). Table 4 summarizes the number of patients with and without DLT in each dose and body weight group.

**TABLE 4:** Patients in opposite DLT groups with 5 and 10 mg doses.

Dose	5 mg		10 mg	
BW (kg)	DLTs (−)	DLTs (+)	DLTs (−)	DLTs (+)
36.1–40.0	1			1
40.1–45.0	1		1	1
45.1–50.0		1	1	2
50.1–55.0	3	1		2
55.1–60.0	2	3		2
60.1–65.0			1	
66.1–70.0			1	

### Association between everolimus exposure and efficacy

**FIGURE 2:**
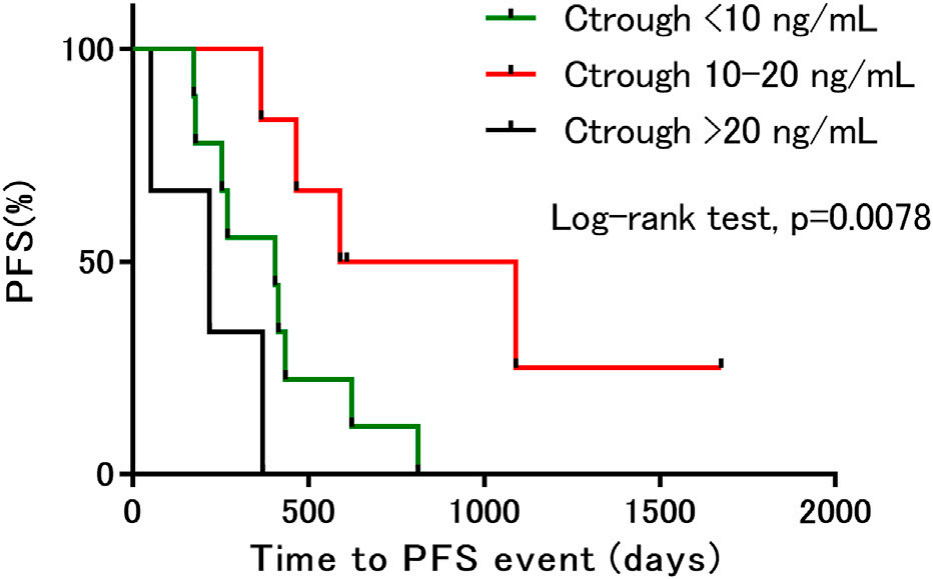
Kaplan–Meier estimates of progression-free survival, based on differences in trough concentration. PFS, progression free survival; C_trough_, trough concentration.

The median PFS in this study population was 13.7 months (range: 1.7–55.8 months). In 14 patients the PFS was more than 8.5 months, which is the median PFS observed in the Japanese population in the BOLERO-2 study (Ito et al., 2015). When the patients were divided into three groups based on the trough concentration ranges <10 ng/ml (*n* = 9), 10–20 ng/ml (*n* = 6), and >20 ng/ml (*n* = 3), we found that PFS was significantly longer in the 10–20 ng/ml group than in the other groups (Figure 2).

### Case

**FIGURE 3:**
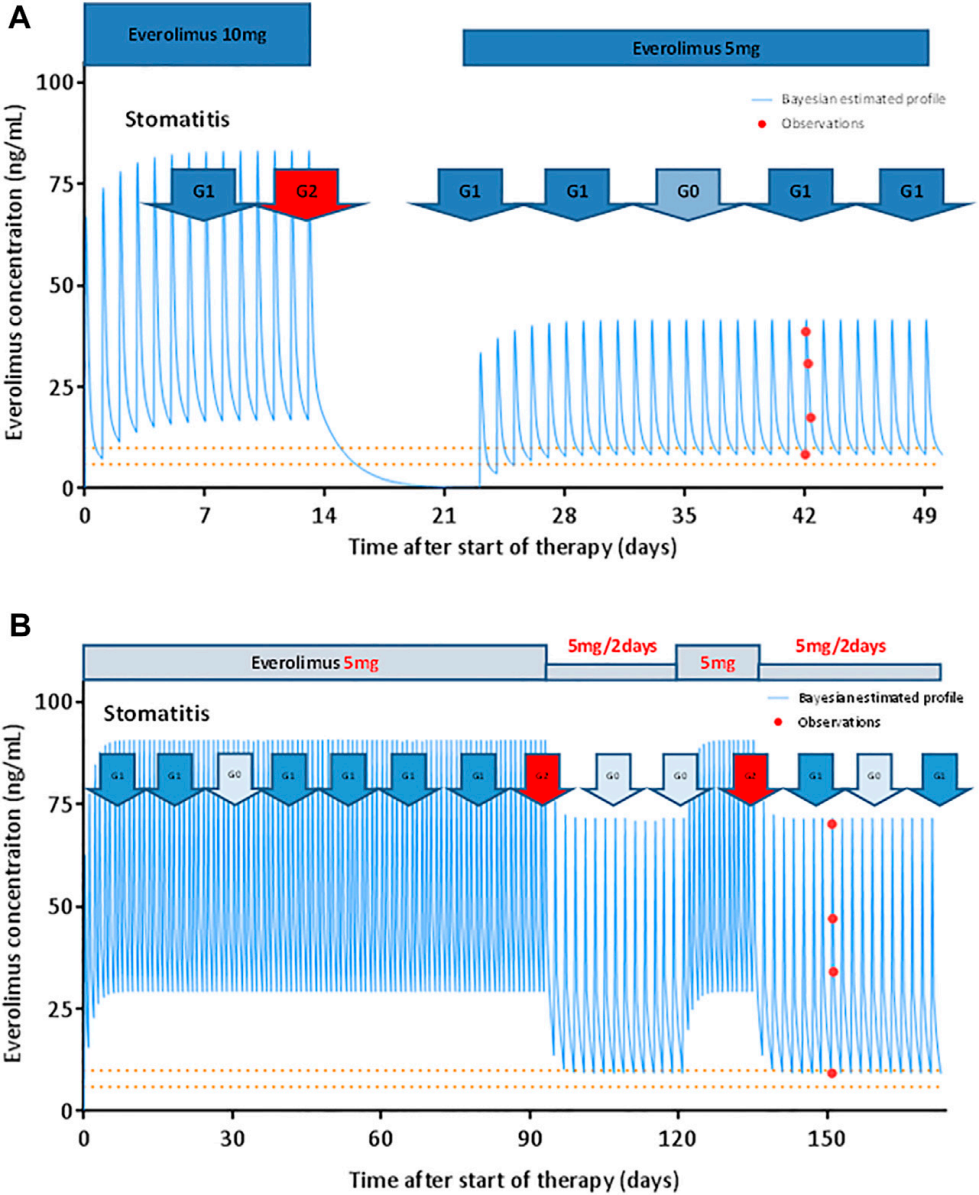
Model-based prediction of everolimus PK profile in representative patients. **(A)** Everolimus starting dose was 10 mg once daily. The patient exhibited grade 2 stomatitis 14 days after treatment initiation. Subsequent doses were suspended for 10 days. After everolimus treatment was resumed at 5 mg once daily, the patient did not show any dose-limiting toxicity. The patient continued the therapy for 8.5 months. The solid line represents the Bayesian estimated everolimus concentration profile. The closed circles represent observed blood concentrations. The dashed lines indicate the target trough concentration range suggested in patients with renal transplant (calcineurin-inhibitor-free regimen). **(B)** Everolimus starting dose was 5 mg once daily. The patient exhibited grade 2 stomatitis 90 days after treatment initiation. The dose of everolimus was reduced to 5 mg every alternate day. The stomatitis subsequently improved, and 28 days later, everolimus was resumed at 5 mg once daily. Two weeks later, the stomatitis worsened to grade 2. The everolimus dose was reduced to 5 mg every alternate day. The patient then progressed with stomatitis grade 0–1 and continued the therapy for 13.8 months.

Figure 3 shows the model-based predicted everolimus PK profile and the observed adverse drug reactions in a representative case. Figure 3A shows a 65-year-old female with multiple recurrent hepatic metastases from breast cancer. The patient started everolimus at 10 mg once daily, as a fourth line therapy. Two weeks after initiation of treatment, she developed grade 2 stomatitis. Therefore, subsequent everolimus doses were withheld. The everolimus trough concentration estimate at this point was 16.8 ng/ml. After 10 days, the stomatitis improved, and administration of everolimus was resumed at 5 mg once daily. The patient continued taking everolimus at that dose without the occurrence of DLTs for the following 7.5 months. The steady-state trough concentration was 8.4 ng/ml after restarting the treatment, and the PFS was 8.5 months.

Figure 3B shows a 64-year-old female patient with multiple recurrent lung and bone metastases from breast cancer. The patient was treated with everolimus at 5 mg once daily, as a fifth-line therapy. Following treatment initiation, stomatitis progressed from grades 0–1 to 2 on day 90. The dose of everolimus was thus reduced to 5 mg every alternate day. The everolimus trough concentration estimate at this point was 29.6 ng/ml. The stomatitis subsequently improved, and 28 days later, everolimus was resumed at 5 mg once daily. Two weeks later, the stomatitis worsened to grade 2; similarly, the everolimus dose was reduced to 5 mg every alternate day. The patients then progressed with stomatitis grade 0–1 and could continue everolimus treatment until PD. The steady-state trough concentration after everolimus dose reduction was 9.2 ng/ml, with PFS at 13.8 months.

## Discussion

In this study, we investigated the characteristics of everolimus PK and the relationship between blood concentration and efficacy as well as adverse events in patients with breast cancer. Inter-individual variation in PK parameters was high in Japanese patients with breast cancer. In addition, the trough concentrations at DLTs were significantly higher than the trough concentrations in the absence of DLTs. To the best of our knowledge, this is the first study to show a therapeutic trough concentration range of everolimus that prolongs PFS in patients with breast cancer.

The BOLERO-2 study reported that the adverse drug reactions of everolimus, such as stomatitis, skin rash, dysgeusia, and non-infectious lung-related adverse events, occurred more frequently in Japanese patients than in other patient populations (Ito et al., 2015). In the present study, a considerable proportion of patients (66% (8/12) on an everolimus starting dose of 10 mg and 83% (5/6) on a starting dose of 5 mg) required dose reduction owing to adverse drug reactions. In addition, 39% (7/18) of all patients required a second dose reduction from the starting dose of 10 mg. These numbers were higher than those reported in another clinical study conducted in Spain, wherein 48% (43/90) of patients required a dose reduction to 5 mg daily and 7% (6/90) of patients required a second dose reduction to 2.5 mg daily (Pascual et al., 2017). These results suggest that there is a high incidence of everolimus adverse drug reactions in Japanese patients with breast cancer, with a considerable number of patients requiring dose reduction.

In this study, three patients received clarithromycin, 200 mg twice daily for 7 days, a CYP3A4 inhibitor, during the everolimus treatment period had no DLT while being treated with clarithromycin. However, Miesner et al. (2016) reported a male kidney cancer patient, treated with 10 mg everolimus, hospitalized for acute renal failure after receiving concomitant clarithromycin 500 mg twice daily for 12 days. The trough level of everolimus was 110 ng/ml. Therefore, caution should be exercised when everolimus is combined with clarithromycin.

In all the patients, the steady-state everolimus trough concentration estimated at the starting dose was higher than the upper limit of the target trough concentration range of 6–10 ng/ml (calcineurin-inhibitor-free regimen) used for kidney transplantation (Shipkova et al., 2016). In addition, the estimated trough concentration after dose reduction was higher than 6 ng/ml, except in one patient. Generally, to suppress rejection after renal transplantation, an everolimus starting dose of 1.5 mg/day is recommended, followed by dose adjustment to reach the target trough value (Lorber et al., 2005; Vítko et al., 2005). However, for patients with breast cancer, everolimus treatment is started at a dose of 10 mg/day, which leads to a higher trough concentration and higher incidence of adverse events than those in renal transplant patients. This indicates that patients with breast cancer who are administered everolimus are in a more immunosuppressed state than patients undergoing everolimus treatment after kidney transplantation.

de Wit et al. (2016) reported that among patients with thyroid cancer, those who needed dose reduction because of the toxicity of everolimus had significantly higher exposure to everolimus than those who did not require dose reduction; moreover, there was a significant relationship between everolimus exposure and stomatitis. In a review of therapeutic drug monitoring of everolimus in cancer, Falkowski and Woillard (2019) reported that everolimus toxicity was significantly associated with high trough concentrations. In our study on advanced and metastatic breast cancer, trough concentrations at which time patients experiencing DLTs were significantly higher than when patients did not have DLTs and maintained everolimus treatment. This suggested that high exposure to everolimus led to toxicity and caused a decrease in tolerability.

Although it was often necessary to reduce the dose of everolimus in the present study, the therapeutic effect of everolimus was sufficiently obtained by adjusting the dose appropriately. In addition, PFS was significantly prolonged in the 10–20 ng/ml trough concentration group. Thus, the therapeutic effect of everolimus could be optimized by measuring the blood concentration of everolimus and adjusting the dose to achieve a target trough concentration within the range of 10–20 ng/ml. However, the median trough concentration in patients with DLTs was 19.0 ng/ml, suggesting that further studies are needed to establish an upper limit of the target trough concentration. Deppenweiler et al. (2017) reported that among the patients with breast, kidney, and neuroendocrine cancer, trough concentrations of everolimus higher than 26.3 ng/ml were associated with a 4-fold increased risk of toxicity, whereas trough concentrations lower than 11.9 ng/ml were associated with a 3-fold increased risk of progression. In a review, Falkowski and Woillard (2019) reported that a trough concentration higher than 20 ng/ml could be proposed as a threshold to indicate an increased risk of overall severe toxicity. Furthermore, Roušarová et al. (2021) reported that a trough concentration between 10 and 20 ng/ml was a promising therapeutic drug monitoring target for everolimus in breast cancer treatment, in a review of therapeutic drug monitoring of protein kinase inhibitors in patients with breast cancer. The findings from these reports support our study’s findings. The study indicates that adjusting the trough concentration of everolimus to 10–20 μg/ml may significantly prolong PFS, but this remains to be confirmed in a larger sample size.

In this study, high inter-individual variation in everolimus PK was observed in Japanese patients with breast cancer. To date, various studies have determined factors associated with everolimus PK. Everolimus is primarily metabolized by CYP3A4 and CYP3A5 (Kirchner et al., 2004; Anglicheau et al., 2007). In renal transplant patients, body weight, age, race, and concomitant administration of erythromycin or azithromycin have been identified as significant covariates predictive of everolimus clearance (Kovarik et al., 2001). In heart transplant patients, total bilirubin and concomitant administration of cyclosporine are associated with everolimus clearance (Lemaitre et al., 2012). In this study, genetic polymorphisms in CYP3A5 and efflux transporter ABCB1 (also known as P-glycoprotein) were also investigated; however, a significant covariate for clearance could not be identified. In patients with advanced recurrent breast cancer treated with everolimus and exemestane, the blood everolimus concentration in patients with the CYP3A4*22 allele was significantly higher than that in patients without the *22 allele (Pascual et al., 2017). The source of the PK variabilities, including the effect of genetic polymorphisms, could not be evaluated in this study owing to limited data availability.

The limitations of this study were a small sample size, single-center design, and the use of different methods to measure the blood concentration of everolimus, which depend on the survey period. However, it was shown that trough concentrations between 10 and 20 ng/ml without DLTs may contribute to the prolongation of PFS. A further study with a larger sample size is warranted to evaluate the findings in this study.

In conclusion, this study characterized the everolimus PK parameters in Japanese patients with breast cancer. The results indicated that exposure to high everolimus concentrations was associated with poor tolerability. Based on our data, trough concentrations between 10 and 20 ng/ml may be associated with prolonged PFS. Determining the blood concentration of everolimus and subsequent dose adjustment will potentially reduce side effects and enhance the therapeutic effect in Japanese patients with advanced breast cancer.

## Data Availability

The original contributions presented in the study are included in the article/Supplementary Material, further inquiries can be directed to the corresponding author.

## Abbreviations

PK, Pharmacokinetics; CL, Clearance; DLTs, Dose-limiting toxicities; PFS, Progression-free survival; Q, intercompartment clearance; V1, volume of distribution of the central compartment; V2, peripheral compartment.
